# Recent Advances of SnO_2_-Based Sensors for Detecting Fault Characteristic Gases Extracted From Power Transformer Oil

**DOI:** 10.3389/fchem.2018.00364

**Published:** 2018-08-29

**Authors:** Qingyan Zhang, Qu Zhou, Zhaorui Lu, Zhijie Wei, Lingna Xu, Yingang Gui

**Affiliations:** ^1^College of Engineering and Technology, Southwest University, Chongqing, China; ^2^Electrical and Computer Engineering Department, Wayne State University, Detroit, MI, United States

**Keywords:** tin oxide, gas sensors, fault characteristic gases, power transformer oil, sensing properties, sensing mechanism

## Abstract

Tin oxide SnO_2_-based gas sensors have been widely used for detecting typical fault characteristic gases extracted from power transformer oil, namely, H_2_, CO, CO_2_, CH_4_, C_2_H_2_, C_2_H_4_, and C_2_H_6_, due to the remarkable advantages of high sensitivity, fast response, long-term stability, and so on. Herein, we present an overview of the recent significant improvement in fabrication and application of high performance SnO_2_-based sensors for detecting these fault characteristic gases. Promising materials for the sensitive and selective detection of each kind of fault characteristic gas have been identified. Meanwhile, the corresponding sensing mechanisms of SnO_2_-based gas sensors of these fault characteristic gases are comprehensively discussed. In the final section of this review, the major challenges and promising developments in this domain are also given.

## Introduction

Power transformers are one of the most important apparatuses in power systems, and their reliability is extremely vital to ensure the stable system operation (Zheng et al., 2018). Thermal and electrical faults in oil-filled power transformers may produce typical fault characteristic gases including hydrogen H_2_ (David et al., 2018), carbon monoxide CO (Joseph, 1980; Uddin et al., 2016; Zhou et al., 2018b), carbon dioxide CO_2_ (2015; Dan et al., 2016; Iwata et al., 2017; Zhang et al., 2017), methane CH_4_ (Sedghi et al., 2010), acetylene C_2_H_2_ (Qi et al., 2008), ethylene C_2_H_4_, and ethane C_2_H_6_. These typical fault characteristic gases could be dissolved in transformer oil or accumulate as free gases if produced rapidly in large quantities. Therefore, detection and analysis of the species, quantities and generation rates of these fault gases presented in the fluid allow for the identification of power transformer fault types such as corona, sparking, overheating, and arcing.

During the past few decades, dissolved gas analysis has been developed to detect the latent faults of oil-immersed power transformers (Morais and Rolim, 2006; Zhan et al., 2015; Uddin et al., 2016; Gong et al., 2018). Gas sensing detection technology is the core of dissolved gas analysis. Different types of sensing technologies have been reported in previous studies for detecting typical fault characteristic gases extracted from transformer oil, such as metal oxide semiconductor gas sensors (Zhu and Zeng, 2017a; Chen et al., 2018), catalytic combustion sensors (Liu et al., 2011), fuel cell sensors (Modjtahedi et al., 2016; Tonezzer et al., 2017), and optical sensors (Trieu-Vuong et al., 2016; Paliwal et al., 2017). Given the remarkable advantages of simple fabrication process, low maintenance cost, fast response and recovery, long service life, and so on, metal oxide semiconductor materials like SnO_2_ (Choi et al., 2014; Zeng et al., 2014), ZnO (Zhou et al., 2017; Zhu and Zeng, 2017b; Zhu et al., 2017), TiO_2_ (Zeng and Liu, 2010; Zhang et al., 2018b) and In_2_O_3_ (Cao et al., 2015) have received scientific and technological importance for many years and are widely used to detect these gases. Among them, as the most widely used gas-sensitive material, SnO_2_ is currently the main sensing materials used in experimental research and commercial application for detecting typical fault characteristic gases extracted from power transformer oil (Zhang et al., 2010; Zhou et al., 2018a).

Herein, the first section of this review will briefly outline the preparation of the currently used SnO_2_ sensing materials, the microstructure morphology, and doping modification of SnO_2_ sensing materials for detecting typical fault characteristic gases extracted from power transformer oil. The second section addresses the main gas sensing mechanisms of SnO_2_ gas sensors for these gases. The third section presents the conclusions, focusing on future challenges and potentialities associated with SnO_2_-based gas sensors for detecting these typical fault characteristic gases.

## Sensing performances of SnO_2_-based sensors to fault characteristic gases extracted from power transformer oil

For detecting and analyzing typical fault characteristic gases extracted from power transformer oil with SnO_2_-based gas sensors, the most important concerns are the sensor sensitivity, selectivity, and repeatability (Fan et al., 2017). Combined with the research concerns mentioned above, this section briefly summarizes recent progress in the application of SnO_2_ sensing materials to detect typical fault characteristic gases dissolved in transformer oil. The gas sensing performance of some modified SnO_2_ gas sensors are listed in Table 1.

**Table 1:** Comparison of the representative SnO_2_ based sensors for fault characteristic gases extracted from power transformer oil.

**Target gas**	**Sensing material**	**Synthesis method**	**Morphology**	**Detection range (ppm)**	**T_oper._(°C)**	**S/ppm**	**Selectivity**	**References**
H_2_	1 wt% Co-doped SnO_2_	Electrospinning	Nanofibers	100–25,000	330	24/100	H_2_ > CO	Liu et al., 2010
	1 mol% Au-doped SnO_2_	Sol-gel	Nanoparticles	1–5,000	400	48/5,000	H_2_>CO	Yin and Tao, 2017
	Pd-decorated SnO_2_	Chemical vapor deposition	Nanowires	10–100	350	7.1/100	H_2_>CO_2_	Nguyen et al., 2017
CO	1.5% Pd-doped SnO_2_	Co-precipitation	Thick-film	100–1,000	260	6.59/400	–	Chen et al., 2018
	ZnO–SnO_2_ nanoparticles	Typical hydrothermal	Nanoparticles	40–160	300	13/100	–	Chen et al., 2015
	Au@SnO_2_	Hydrothermal deposition	Yolk–shell nanospheres	5–100	210	30/50	CO>H_2_>C_2_H_4_>CO_2_	Wang et al., 2013
CO_2_	LaFeO_3_/SnO_2_	Sol-gel	Thick film	–	250	2.72/4,000	–	Zhang et al., 2017
	LaOCl-doped SnO_2_	One-step electrospinning	Nanofibers	100–20,000	300	3.7/1,000	–	Xiong et al., 2017
CH_4_	20 mol% Pt-SnO_2_	Electrospinning	Nanofibers	1–1,000	350	1.11/1	–	Lu et al., 2018
	Pt-SnO_2_	Wet chemical	Thin film	1000–10,000	400	1.55/1,000	–	Min and Choi, 2005
C_2_H_2_	Sm_2_O_3_-doped SnO_2_	Sol–gel	Nanoparticles	1–5,000	180	63.8/1,000	C_2_H_2_>CO>CH_4_>H_2_	Qi et al., 2008
	rGO-Loaded SnO_2_	Hydrothermal	Nanoparticles	0.5–500	180	12.4/50	C_2_H_2_>CH_4_>H_2_>CO>CO_2_	Jin et al., 2017
C_2_H_4_	silicalite-1 layer coated on SnO_2_	Ultrasonic spray pyrolysis technique	Thin film	2–70	350	2.21/8	–	Jadsadapattarakul et al., 2010
	SnO_2_	R.F. magnetron sputtering	Thin film	10–100	300	10.43/100	–	Ahn et al., 2010
C_2_H_6_	5 wt% Pd-doped SnO_2_	Hydrothermal	Nanoparticles	5–100	400	5.89/100	–	Chen et al., 2013a

T_oper_, temperature at which the best sensor performance, usually in terms of the highest response toward target gas, could be obtained.

S, gas sensing response of a SnO_2_-based sensor to target gas, which is defined as S = Ra/Rg for reducing gas and *S* = Rg/Ra for oxidizing gas (where Ra and Rg are the resistance of the sensor in air and in the test gas, respectively).

It is known that the metal oxide semiconductor SnO_2_ is a promising material for sensing the typical fault characteristic gases extracted from power transformer oil. Nevertheless, the sensing performance of the proposed sensors considerably depends on the preparation method and surface structure of the sensing materials and the dopants. The structure of modern SnO_2_ gas sensors could be classified into two main types including thick/thin films and nanoparticles. The preparation methods of the sensing materials mainly include the hydrothermal method, the sol-gel method, the electrospinning technique, chemical vapor deposition and so on. Different preparation methods could affect the morphology of the sensing materials and further change their gas sensing properties (Long et al., 2018; Zhang et al., 2018a; Zhou et al., 2018c). Noble metals or metal oxide doped on SnO_2_-based sensors can play an important role in accelerating the sensing process and improving the gas sensor performances.

In power transformers, H_2_ is generated from the thermal decomposition of oil at high temperatures, which is a serious problem for transformer oil (Uddin et al., 2016). Various high-efficacy and workable SnO_2_-based sensors have been recently introduced for detecting H_2_. It can be seen from the data in Table 1 that various metal particles such as Co (Liu et al., 2010), Au (Yin and Tao, 2017), and Pd (Nguyen et al., 2017) have been added to SnO_2_-based sensors to enhance H_2_ gas sensing performance. Among them, the Au-loaded SnO_2_ sensor can detect H_2_ down to 1 ppm, which is a good property for detecting low concentrations of H_2_ dissolved in transformer oil (Yin and Tao, 2017). K. Inyawilert reported that a SnO_2_ sensing film with an optimal Rh-doping level of 0.2 wt% exhibited an ultra-high response of 22,170 and a short response time of 6 s toward 30,000 ppm H_2_ at an optimum operating temperature of 300°C. In addition, the proposed Rh-doped SnO_2_ sensor displayed good H_2_ selectivity against NO_2_, SO_2_, C_2_H_4_, C_3_H_6_O, CH_4_, H_2_S, and CO (Inyawilert et al., 2017). Except for nanoparticles embedded in the metal oxide matrix, the morphology of the SnO_2_ materials could be applied to improve the H_2_ sensing properties, and low dimensional nanostructures have attracted increasing attention. Nguyen Kien et al. introduced the on-chip growth of SnO_2_ nanowire-based gas sensors to detect low concentrations of H_2_ gas, which responded with 2.6 for 20 ppm H_2_ gas at 400°C (Nguyen et al., 2017). Taken together, these findings suggest that the Au-loaded SnO_2_ gas sensor is the most promising candidate for detecting low concentration H_2_ extracted from power transformer oil.

The insulating paper, pressboard and wood blocks in power transformers contain a large number of anhydroglucose rings as well as weak C-O molecular bonds and glycosidic bonds that are thermally less stable than the hydrocarbon bonds in oil. These bonds could decompose to CO and CO_2_ at lower temperatures when potential faults occur. As shown in Table 1, Pt-doped SnO_2_ thick film (Chen et al., 2018), ZnO-SnO_2_ nanoparticles (Chen et al., 2015), and Au-doped SnO_2_ yolk-shell nanospheres (Wang et al., 2013) could be potential candidates for CO sensors. Among them, Wang et al. compared the gas sensing properties of Au@SnO_2_ yolk-shell nanospheres with that of hollow SnO_2_ nanospheres. The sensor fabricated with the Au@SnO_2_ yolk-shell nanospheres showed lower operating temperature (210°C), lower detection limit (5 ppm), faster response (0.3 s), and better selectivity to CO gas (Wang et al., 2013), which could be attributed to the catalytic effect of Au and enhanced electron depletion at the surface of the Au@SnO_2_ yolk–shell nanospheres. Moreover, SnO_2_ loaded with Pd nanoparticles may be another kind of promising material for CO gas sensing (Zhou et al., 2018d). Yuasa et al. prepared PdO-loaded SnO_2_ nanoparticles by the reverse micelle method and reported that the 0.1 mol% PdO-loaded SnO_2_ sensor exhibited a high gas response value of 320 to 200 ppm CO gas (Yuasa et al., 2009). Chen et al. synthesized Pd-doped SnO_2_ nanoparticles using a co-precipitation method, and the 1.5 wt% PdO decorated SnO_2_ presented the largest gas-sensitive response of 6.59 at 260°C in 400 ppm CO atmosphere (Chen et al., 2018). Another study reported that Pd-modified nanocrystalline SnO_2_ displayed a fairly high and reversible CO response (2–100 ppm) at room temperature (Marikutsa et al., 2010). Yin et al. prepared Pd-loaded and Fe-doped SnO_2_ by the sol-gel method. The composite with 10 mol% Fe and 0.2 mol% Pd had the highest sensitivity and selectivity to CO in the range of 200–3,000 ppm at 350°C, and the response value to 2,000 ppm CO was increased 13 times compared with pure SnO_2_ (Yin and Guo, 2014). Therefore, Pd is a highly effective catalyst for improving the sensing performance of SnO_2_-based sensors to CO gas (Xiong et al., 2017).

Due to their high chemical stability, conventional binary metal oxides have very low sensitivity to chemically inert gases such as CO_2_ (Korotcenkov and Cho, 2017). However, it was reported that La-doped SnO_2_ nanocomposites can be used for CO_2_ sensing (Kim et al., 2000). And the 8% LaOCl-SnO_2_ nanofibers exhibited an optimal response of 3.7 toward 1,000 ppm CO_2_ at 300°C with response/recovery times of 24/92 s (Xiong et al., 2017). Karthik et al. reported the sensing properties of tin oxide (SnO_2_) and zinc oxide (ZnO) thin films deposited onto macroporous silicon (PS) substrates to CO_2_ and found that the obtained SnO_2_/PS films showed the highest sensing response of 19 to 15 ppm CO_2_ gas (Karthik et al., 2018).

For hydrocarbons, namely, CH_4_, C_2_H_2_, C_2_H_4_, and C_2_H_6_, when high energy discharge occurs, such as arcing, in transformer oil, the content of C_2_H_2_ is relatively high. At low temperature thermal faults (*T* < 300°C), the contents of CH_4_ and C_2_H_6_ tend to be high. C_2_H_4_ is the main content of hydrocarbon while at high temperature thermal faults (*T* > 300°C) (Fan et al., 2017). For detecting these hydrocarbons, an online monitoring system based on a SnO_2_-based gas chromatographic detector for assessing the running condition of a power transformer was developed (Fan et al., 2017). Qi et al. fabricated a C_2_H_2_ sensor based on 6 wt% Sm_2_O_3_-doped SnO_2_, whose gas response to 1,000 ppm C_2_H_2_ could reach 63.8, 16.8 times larger than that of pure SnO_2_ (Qi et al., 2008). Moreover, Jin et al. reported that reduced graphene oxide (rGO)-loaded SnO_2_ hybrid nanocomposite showed high sensor response (12.4 toward 50 ppm), fast response-recovery time (54 and 23 s), low detection limit (1.3 ppm), good linearity, excellent selectivity and long-term stability to C_2_H_2_ (Jin et al., 2017). These results indicated that rGO would be an effective addition to enhance the sensing properties of SnO_2_-based sensors to C_2_H_2_ and make a contribution to developing a ppm-level gas sensor for on-line monitoring of C_2_H_2_ gas extracted from transformer oil.

Sensors for CH_4_ detection have also been widely studied and are partly summarized in Table 1. The 20 mol% Pt-SnO_2_ nanofibers exhibited excellent CH_4_ sensing properties over a temperature range of 100–350°C, and an obvious response of 1.11 to 1 ppm CH_4_ was measured at 350°C (Lu et al., 2018). Koeck et al. found a device prepared by ultra-long single crystalline SnO_2_-nanowires, which was able to detect a few ppm of CO and CH_4_ at the operating temperature of 200–250°C (Koeck et al., 2009). Chen et al. successfully synthesized Co-doped SnO_2_ nanofibers via an electrospinning method and reported that the Co-doped SnO_2_ nanofiber sensor exhibited a high response of 30.28 toward 50 ppm CH_4_ at 300°C (Chen et al., 2013b). For C_2_H_4_ and C_2_H_6_ detection, only a few sensors including SnO_2_ thin films and Pd-doped SnO_2_ nanoparticles are found to be effective. Jadsadapattarakul et al. reported that the sensing response, response and recovery time of SnO_2_ thin film sensors for selective detecting of C_2_H_4_ gas could be improved by coating a layer of [010] highly preferred-orientation silicalite-1 polycrystals (Jadsadapattarakul et al., 2010). Ahn et al. obtained SnO_2_ thin films by R.F. magnetron sputtering to fabricate high performance C_2_H_4_ gas sensors (Ahn et al., 2010). Chen et al. discovered that the 5 wt% Pd-doped SnO_2_ sensor could detect C_2_H_6_ at 400°C, and the sensor exhibited the largest gas-sensitive response of 5.89 toward 100 ppm C_2_H_6_ (Chen et al., 2013a).

## Sensing mechanisms of SnO_2_-based sensors to fault characteristic gases extracted from power transformer oil

It is well agreed that the sensing mechanism of SnO_2_ gas sensors is the change in conductivity of the metal oxide layer caused by the interaction with the surrounding atmosphere (Ducéré et al., 2012; Zeng et al., 2013; Korotcenkov and Cho, 2017). When exposed to air, oxygen molecules would be adsorbed on the surface of the SnO_2_ nanostructures and capture electrons from the conduction band of SnO_2_ to generate chemisorbed oxygen species (O^−^, O^2−^, and ${\text{O}}_{2}^{-}$, depending on temperatures) (Shahabuddin et al., 2017). At low temperatures (below 150°C), oxygen molecules exist in the form of molecular ions ${\text{O}}_{2}^{-}$, which would change to atomic ions O^−^ (150–400°C) and O^2−^ (more than 400°C) as temperatures rising (Punginsang et al., 2017). The chemical adsorption process can be explained by the following reactions:

(1) $$\begin{array}{c} O_{2}{}_{\left(\text{gas}\right)}\Leftrightarrow O_{2}{}_{\left(\text{abs}\right)} \end{array}$$

(2) $$O_{2}{}_{\left(\text{abs}\right)}+e^{-}\Leftrightarrow O_{2}{}^{-}(\text{abs})$$

(3) $$O_{2}{}^{-}\left(\text{abs}\right)+e^{-}\Leftrightarrow 2O^{-}{}_{\left(\text{abs}\right)}$$

(4) $$O^{-}{}_{\left(\text{abs}\right)}+e^{-}\Leftrightarrow O^{2-}{}_{\left(\text{abs}\right)}$$

As the electrons transfer from the conduction band of SnO_2_ to the chemisorbed oxygen, the electron concentration and electrical conductivity of the SnO_2_ film decrease. When the SnO_2_ film is exposed to typical fault characteristic gases, the reducing gas would react with the chemisorbed oxygen species, thereby releasing electrons back to the conduction band with increasing electrical conductivity. The sensing mechanisms of the SnO_2_ sensor sensing these fault gases can be explained from the following reaction paths, where O^−^ is taken as an example (Samerjai et al., 2012).

(5) $$\begin{array}{c} \begin{array}{c} {\text{H}}_{2}+{\text{O}}^{{}^{-}}{}_{\left(\text{ads}\right)}\to {\text{H}}_{2}\text{O }+{\text{e}}^{{}^{-}} \end{array} \end{array}$$

(6) $$\begin{array}{c} \begin{array}{c} {\text{H}}_{2}+{\text{O}}^{{}^{-}}{}_{\left(\text{ads}\right)}\to {\text{H}}_{2}\text{O }+{\text{e}}^{{}^{-}} \end{array} \end{array}$$

(7) $$\begin{array}{c} {\text{C}}_{2}{\text{H}}_{2}+{\text{O}}^{-}{}_{\left(\text{ads}\right)}\to 2\text{C }+{\text{H}}_{2}\text{O }+{\text{e}}^{-}+\text{ heat} \end{array}$$

(8) $$\begin{array}{c} {\text{CH}}_{4}+\;2{\text{O}}^{-}{}_{\left(\text{ads}\right)}\to 2{\text{H}}_{2}\text{O }+{\text{ CO}}_{2}+\;2{\text{e}}^{-} \end{array}$$

(9) $${\text{C}}_{2}{\text{H}}_{4}+\;2{\text{O}}^{-}{}_{\left(\text{ads}\right)}\to 2{\text{H}}_{2}\text{O }+\;2\text{C }+\;2{\text{e}}^{-}$$

(10) $$\begin{array}{c} \begin{array}{c} {\text{C}}_{2}{\text{H}}_{6}+\;3{\text{O}}^{-}{}_{\left(\text{ads}\right)}\to 3{\text{H}}_{2}\text{O }+\;2\text{C }+\;3{\text{e}}^{-} \end{array} \end{array}$$

Dopants added to SnO_2_-based gas sensors can accelerate the reaction process mentioned above and improve the sensing performance of gas sensors. The doped catalysts can enhance the sensing performance of a sensor in two ways, namely, chemical sensitization and electronic sensitization (Lin et al., 2017). The high surface area structures such as porous structures and hollow structures could accelerate gas diffusion on the material surface and then shorten the response time.

On the other hand, to understand the sensing mechanism of SnO_2_-based materials at the atomic and quantum levels, based on the framework of Density Function Theory (DFT), Li et al. established a SnO_2_ surface model, gas molecular models and adsorption models. A first principles calculation using the Cambridge Sequential Total Energy Package (CASTEP) program was performed, and the total energies, electronic structures and adsorption properties were investigated in detail. Theoretical calculations provided a qualitative explanation of the sensing properties of the fabricated SnO_2_-based sensor to various fault characteristic gases (CH_4_, C_2_H_6_, C_2_H_4_, and C_2_H_2_) extracted from power transformers (Li et al., 2017). Zeng et al. performed a first principles calculation to investigate how H_2_ gas interacts with the SnO_2_ (110) surface and the effect of metallic ions on the gas response of SnO_2_. Based on the theoretical calculation, it was reported that the Pd-doped SnO_2_ (110) surface could adsorb more H_2_ gas and receive larger electrons from adsorbed H_2_ molecules (Zeng et al., 2011). Other research involving a possible CO sensing mechanism for Pd-doped SnO_2_ sensors was investigated with first principles calculations (Chen et al., 2018), and the theoretical results demonstrated that CO molecules can grab O from the pre-adsorbed oxygen on the Pd^4^ cluster or the PdO cluster on the SnO_2_ (110) surface. These processes may play an important role in CO sensing for Pd-doped SnO_2_ (Chen et al., 2018). In particular, theoretical calculations indicated that CO_2_ molecules cannot be adsorbed onto the stoichiometric SnO_2_ (110) surface or SnO_2_ (110) surface pre-adsorbed by ${\text{O}}_{2}^{-}$ and O^−^ in dry air. However in wet air, CO_2_ could react with O of pre-adsorbed OH^−^, bringing about the formation of carbonates containing (CO_3_)^2−^ and the dissociation/movement of surface OH^−^ groups, accompanying the release of electrons from CO_2_ to the SnO_2_ surface (Wang et al., 2016).

## Conclusion and perspective

In this mini-review, SnO_2_-based gas sensors for detecting typical fault characteristic gases extracted from power transformer oil have been briefly summarized. The analysis shows that the detection performances of SnO_2_-based gas sensors can be obviously enhanced with dopant addition or increasing the surface area of the sensing materials. Despite achieving good results, there is still much room for further development. First of all, due to the cross-sensitivity between these fault characteristic gases, selectivity is a large challenge for developing high performance SnO_2_-based sensors to detect these gases independently. In the future, to promote engineering applications, a combination of SnO_2_-based gas sensors and gas chromatography should be further studied for multi-component detection of these gases. For further development of the gas-sensing performances, the high surface area structures such as hollow and hierarchical nanostructures could be prepared to obtain more active sites for gas diffusion, and different types of dopant elements should be examined. Furthermore, the gas sensing mechanisms are still imperfect and controversial, which cannot effectively guide development of novel SnO_2_-based sensors and limit the prevailing application of these sensors to fault characteristic gases extracted from power transformer oil. Further research should focus on determining a satisfying mechanism model of SnO_2_-based sensors to provide a guide for future work.

## Author contributions

All authors listed have made substantial, direct and intellectual contributions to the work and approved it for publication.

### Conflict of interest statement

The authors declare that the research was conducted in the absence of any commercial or financial relationships that could be construed as a potential conflict of interest.
